# Disruption of zebrafish cyclin G-associated kinase (GAK) function impairs the expression of Notch-dependent genes during neurogenesis and causes defects in neuronal development

**DOI:** 10.1186/1471-213X-10-7

**Published:** 2010-01-18

**Authors:** Ting Bai, Jamie L Seebald, Kyu-Eui Kim, Hong-Mei Ding, Daniel P Szeto, Henry C Chang

**Affiliations:** 1 Department of Biological Sciences, Purdue University, 915 W. State St., West Lafayette, Indiana 47907-2054, USA

## Abstract

**Background:**

The J-domain-containing protein auxilin, a critical regulator in clathrin-mediated transport, has been implicated in *Drosophila *Notch signaling. To ask if this role of auxilin is conserved and whether auxilin has additional roles in development, we have investigated the functions of auxilin orthologs in zebrafish.

**Results:**

Like mammals, zebrafish has two distinct auxilin-like molecules, auxilin and cyclin **G-a**ssociated **k**inase (GAK), differing in their domain structures and expression patterns. Both zebrafish auxilin and GAK can functionally substitute for the *Drosophila *auxilin, suggesting that they have overlapping molecular functions. Still, they are not completely redundant, as morpholino-mediated knockdown of the ubiquitously expressed GAK alone can increase the specification of neuronal cells, a known Notch-dependent process, and decrease the expression of *Her4*, a Notch target gene. Furthermore, inhibition of GAK function caused an elevated level of apoptosis in neural tissues, resulting in severe degeneration of neural structures.

**Conclusion:**

In support of the notion that endocytosis plays important roles in Notch signaling, inhibition of zebrafish GAK function affects embryonic neuronal cell specification and *Her4 *expression. In addition, our analysis suggests that zebrafish GAK has at least two functions during the development of neural tissues: an early Notch-dependent role in neuronal patterning and a late role in maintaining the survival of neural cells.

## Background

The conserved Notch pathway participates in diverse aspects of animal development, and has been implicated in human diseases and cancers [1-3]. Notch encodes a transmembrane receptor, which, upon ligand binding, undergoes proteolytic processing and releases an intracellular fragment capable of acting as a transcription co-regulator. As both Notch and its ligands (also transmembrane proteins) are widely expressed, their activities need to be tightly regulated. One such important regulation appears to be ligand internalization, which plays a critical role in activating Notch receptors [4,5].

Notch ligand internalization utilizes an ubiquitin-dependent endocytic pathway, as two structurally unrelated E3 ubiquitin ligases, *neuralized (neur) *and *mind bomb (dMib)*, can append ubiquitin to DSL (Delta, Serrate, Lag2) ligands [6-13]. Epsin/*lqf (liquid facets) *then recruits the ubiquitinated DSL ligands into clathrin-coated vesicles (CCVs) [14-18]. The scission of these ligand-containing CCVs from the plasma membrane seems critical for Notch activation, as disruption of dynamin function also causes a *Notch*-like defect [4,19,20].

Another relevant factor in *Drosophila *Notch ligand endocytosis is the J-domain protein auxilin [21,22]. First identified in mammals, auxilin is known to cooperate with Hsc70 in mediating the disassembly of clathrin triskelia and coat proteins from newly formed CCVs *in vitro *[23]. The mammalian genomes contain two distinct auxilin-related genes: *auxilin *and *GAK*, differing in the presence of an Ark (**a**ctin-**r**elated **k**inase) family kinase domain and their tissue distributions [23-25]. GAK contains the Ark domain at the N-terminus and it is ubiquitously expressed. In contrast, auxilin lacks the kinase domain and its expression appears to be neuronal. However, the expression of auxilin in non-neuronal cells has recently been demonstrated [26]. Besides uncoating, several other functions during endocytosis have recently been suggested for auxilin family proteins, including facilitating clathrin exchange during coated-pit formation [27,28], participating in pit constriction [29] and preventing precipitous assembly of clathrin cages [26,30]. Furthermore, GAK has been implicated in clathrin-mediated trafficking from the trans Golgi network (TGN) [25,31].

While it is unclear which of the aforementioned cellular functions are most relevant under physiological conditions, mutations in the sole *Drosophila *auxilin ortholog (*dAux*) clearly disrupt several Notch-dependent processes [21,22]. One such process is the patterning of neural tissues, in which the cells destined to become neurons send a Notch-mediated signal to prevent neighboring cells from adopting neuronal fate. In the absence of this lateral inhibition, supernumerary neuronal cells are generated, forming the so-called neurogenic phenotype [2]. Consistent with the notion that *dAux *participates in Notch signaling, excessive neurons were seen in both the embryonic CNS and the larval eye discs of *dAux *mutants [21,32]. Mosaic analysis showed that the function of dAux during Notch signaling is required in the signal-sending cells, suggesting that it has a role in ligand internalization [32,33].

Given the high degree of conservation of the Notch pathway, it seems reasonable to expect an inhibition of auxilin function in other animal systems would cause *Notch*-like defects. However, as multiple distinct endocytic pathways exist in higher metazoans [34], it is possible that different endocytic pathways can functionally substitute for one another in Notch ligand endocytosis. Inhibition of auxilin function by RNA interference in *C. elegans *was shown to disrupt the receptor-mediated uptake of yolk proteins, yet no Notch-related defects were reported [35]. RNAi-mediated reduction of GAK function in mammalian cells appeared to deregulate EGF signaling and promote tumorigenesis [36]. Tissue-specific inactivation of mouse *GAK *during embryonic development caused severe degenerations in brain, liver, and skin [37], but it is unclear whether any of these defects were due to a disruption of Notch function. Thus, while it seems clear that auxilin family proteins are important for animal viability, whether their function in Notch ligand endocytosis is evolutionarily conserved requires additional investigations.

We have used zebrafish to further assess the roles of auxilin-dependent endocytosis in animal development. Besides being a versatile model organism, zebrafish is suitable for our purpose because the importance of Notch ligand endocytosis has been demonstrated during embryonic neural patterning [9]. We show that zebrafish, like mammals but unlike *Drosophila*, contains both *auxilin *and *GAK*. Zebrafish *auxilin *and *GAK *are interchangeable in their abilities to substitute for *dAux *during *Drosophila *Notch signaling, suggesting that they share some cellular functions. However, they have different expression patterns during development, suggesting that these two paralogs are not completely redundant. Morpholino-mediated knockdown of *GAK *function during embryogenesis caused an increase in the formation of neuronal cells and a decrease in the expression of a Notch target gene, supporting our hypothesis that the role of auxilin family proteins in Notch signaling is conserved. Furthermore, we showed that embryos deficient in GAK function had a higher level of programmed cell death in neural tissues, suggesting that GAK is required for the survival of neuronal cells.

## Results

### The zebrafish genome contains both *GAK *and *auxilin*

To examine the roles of auxilin-related genes during vertebrate development, we first sought to identify *auxilin *or *GAK *in zebrafish genome. Database search revealed that, like mammals, the zebrafish genome contains both *GAK *(*zGAK*, XP_001919224) and *auxilin *(*zAux*, XP_001336673) orthologs, located on chromosome 23 and 6 respectively. This presence of two distinct auxilin-related orthologs appears to be a feature shared by other vertebrates, as the chicken (*Gallus gallus*, XP_424873 and XP_422527) and pufferfish (*Tetraodon nigroviridis*, CAG08624 and CAG11595) genomes both contain *GAK *and *auxilin*. In contrast, arthropods such as *Drosophila *(*melanogaster *and *virilis*, XP_002058717), honeybee (*Apis mellifera*, XP_396906) and flour beetle (*Tribolium castaneum*, XP_967193) have only GAK. *C. elegans *also has only one *auxilin*-related gene, but it lacks both the kinase and the PTEN homologous region [35].

**Figure 1:**
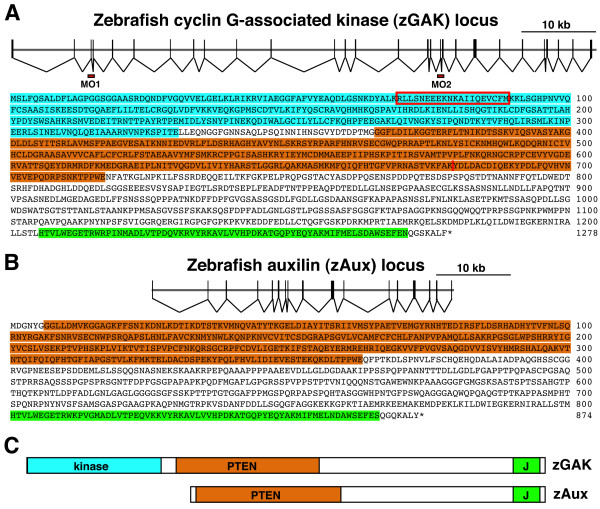
**The genomic organizations and the protein sequences of zebrafish GAK and auxilin**. (A) The *zGAK *locus contains 28 exons and spans 78 kb of genomic DNA. The corresponding cDNA contains a 3834 bp ORF, encoding a polypeptide of 1278 amino acids with an Ark kinase domain (blue), a PTEN-homologous region (orange), a clathrin-binding motif (CBM), and a J-domain (green). Two morpholinos, GAK-MO1 and GAK-MO2, (red boxes) were designed to disrupt the splicing of *zGAK *mRNA by blocking the splice acceptors of exon3 and exon19, respectively. The amino acids deleted by GAK-MO1 injection are boxed in red. The premature stop codon (after Lys679) generated by GAK-MO2 injection is indicated by a red line. (B) The *zAux *locus is smaller, as it contains 17 exons and spans across 40 kb of genomic DNA. The zAux protein, encoded by a 2619 bp ORF, contains a PTEN-related region (orange), CBM, and J-domain (green). (C) A schematic drawing comparing the domain composition of zebrafish auxilin-related genes. The kinase, PTEN, and the J-domains are represented by blue, orange, and green boxes, respectively.

The *zGAK *locus encodes a polypeptide of 1278 amino acids and, like other GAK orthologs, contains an N-terminal Ark kinase, a PTEN domain, a CBM region and a C-terminal J domain (Figure 1A). Alignment of protein sequences shows that zGAK is 65% identical to human GAK overall, with the sequence conservations concentrated in the kinase, PTEN and the J-domain. The kinase, PTEN and J-domain of zGAK are 82.4%, 84.7%, and 78.8% identical to the corresponding domains of human GAK respectively. In contrast, the CBM domains of zGAK and human GAK are more divergent, sharing only 31.7% amino acid sequence identity.

**Figure 2:**
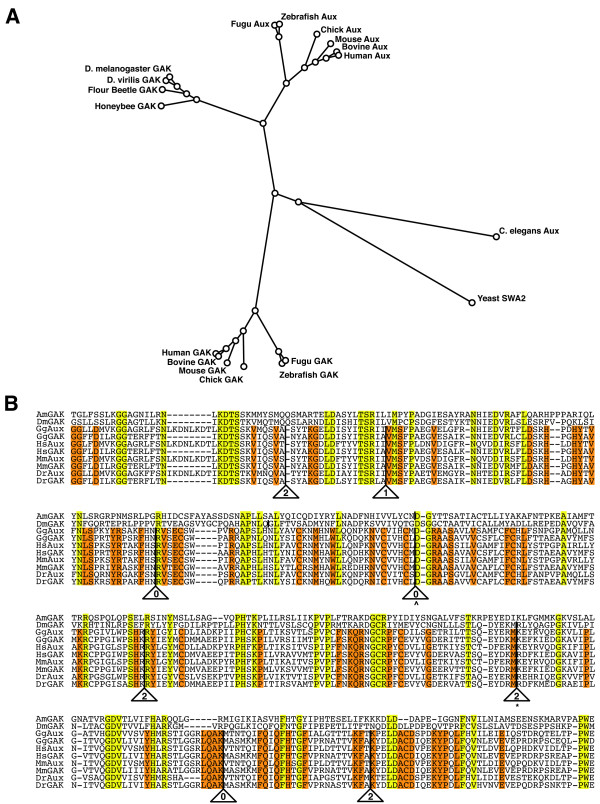
***zGAK *and *zAux *arose from gene duplication**. (A) A phylogenetic analysis of the amino acid sequences of auxilins and GAKs without their kinase domains. The alignment was performed using Geneious software (Biomatters). (B) Alignment of intron positions in the PTEN region of auxilin and GAK orthologs. The residues shared by all are shaded in yellow and the residues conserved in mammals and vertebrates are shaded in orange. The presence of an intron is indicated by a solid line, and the phase is indicated by a number inside the triangle. ^ indicates an intron that is conserved in honeybee but not in *Drosophila*, whereas * indicates an intron that is conserved in fly but not in honeybee. Am: *Apis mellifera*, Dm: *Drosophila melanogaster*, Gg: *Gallus gallus*, Hs: *Homo sapiens*, Mm: *Mus musculus*, and Dr: *Danio rerio*.

The zAux protein, on the other hand, is an 873 amino acid long polypeptide, which contains a PTEN region, a CBM and a J-domain, but lacks the N-terminal kinase (Figure 1B). The protein sequence of zAux is 59.3% identical overall to the human auxilin. Again, the conservations are higher in the PTEN and the J-domain, as these domains share 67.5% and 94.5% amino acid identity respectively. These domains of zAux are also highly related to the PTEN and the J-domain of zGAK (50.8% and 89.4% protein sequence identity respectively), suggesting that *zAux *and *zGAK *are derived from a common ancestral gene. However, while zAux is similar to zGAK, it is more similar to human and mouse auxilin proteins (Figure 2A), suggesting that the divergence of GAK and auxilin occurred prior to the divergence of fish and mammals during vertebrate evolution.

In addition to the similarities in protein sequences, the intron/exon organizations among *auxilin *family genes are highly conserved. First, the human, mouse, and zebrafish *GAK *loci all contain an identical number (27) of introns. The *zAux *locus appears to have lost one intron in the CBM during evolution, as it contains 16 introns while the human and mouse auxilins have 17. Furthermore, in the conserved domains, the positions and the phases (0, 1, or 2 in the codons) of these introns can be precisely aligned. For example, the PTEN homologous regions of zebrafish, mouse, and human *GAK *and *auxilin *loci all contain 8 introns (Figure 2B). These introns, although different in nucleotide lengths, are located at the same positions in the coding region with identical phases. This high degree of conservation in intron/exon organization strongly bolsters the notion that *GAK *and *auxilin *were derived from a common ancestral gene through gene duplication.

### Overexpression of zGAK or zAux causes clathrin aggregations

To ask if zebrafish auxilin family proteins have similar cellular functions as their respective mammalian counterparts, we determined the subcellular localizations of zGAK and zAux. Both *zGAK *and *zAux *were tagged with GFP at the N-termini, placed under the control of a *CMV *promoter in *pCS2*, and transiently expressed in HeLa cells. These GFP-tagged fusion proteins are functional, as ectopic expression of either GFP-zGAK or GFP-zAux in *Drosophila *could restore the neurogenic defect and the lethality caused by *dAux *mutations (see below). To reveal clathrin-positive structures, these cells were also stained with a mouse monoclonal antibody against clathrin heavy chain.

**Figure 3:**
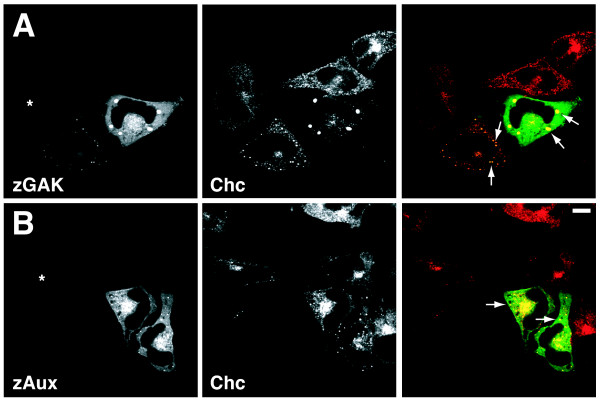
**The subcellular distributions of zebrafish auxilin family proteins**. Spinning disc confocal micrographs of HeLa cells transfected with (A) pCS2-GFP-zGAK and (B) pCS2-GFP-zAux. The cells were also stained for clathrin heavy chain (red). The low GFP-expressing cells are indicated by asterisks. The large GFP- and clathrin-positive aggregates are indicated by arrows. Scale Bar, 10 μm.

As shown in Figure 3, qualitatively different GFP patterns were seen in *pCS2-GFP-zAux *and *pCS2-GFP-zGAK *transfected cells, depending on the expression levels. In cells expressing low levels of GFP-zGAK or GFP-zAux (marked by asterisks), GFP signals were mostly cytosolic and slightly enriched near the perinuclear regions. These perinuclear zGAK- and zAux-positive structures showed overlaps with clathrin, most likely representing the TGN.

In cells expressing high levels of GFP-zGAK and GFP-zAux, the cytosolic staining and the enrichments near the perinuclear regions could still be seen. However, these cells also contained large GFP-positive aggregates (indicated by solid arrows and arrowheads) that were intensely clathrin-positive. In untransfected cells, clathrin staining had a vesicular appearance throughout the cytosol, but it appeared depleted from these structures in those aggregate-containing cells. We noticed that zGAK seemed more capable of causing these aggregates than zAux, as intense GFP-positive and clathrin-containing structures were readily seen in cells expressing milder levels of GFP-zGAK. It has been shown that endogenous GAK is predominantly cytosolic and shows elevated associations with TGN [24,25,31], and over-expression of mammalian auxilin family proteins in HeLa cells can cause the formation of clathrin-containing "granules" [24,38]. Thus, our results showed that over-expressed zGAK and zAux are localized similarly within the cells. In addition, their localizations are similar to those of their respective mammalian homologs.

### zGAK and zAux are functionally interchangeable in rescuing *dAux *defects

To determine whether there are intrinsic functional differences between zGAK and zAux, we compared their abilities in rescuing the extra photoreceptor defect and the lethality caused by *dAux *mutations. N-terminally GFP-tagged *zGAK *and *zAux *were placed under *UAS *control, and transgenic flies carrying these constructs were generated. We reasoned that if zGAK and zAux function similarly during clathrin-mediated transport, both should be able to supplant dAux function during neuronal differentiation in *Drosophila *eye discs and for animal survival.

**Figure 4:**
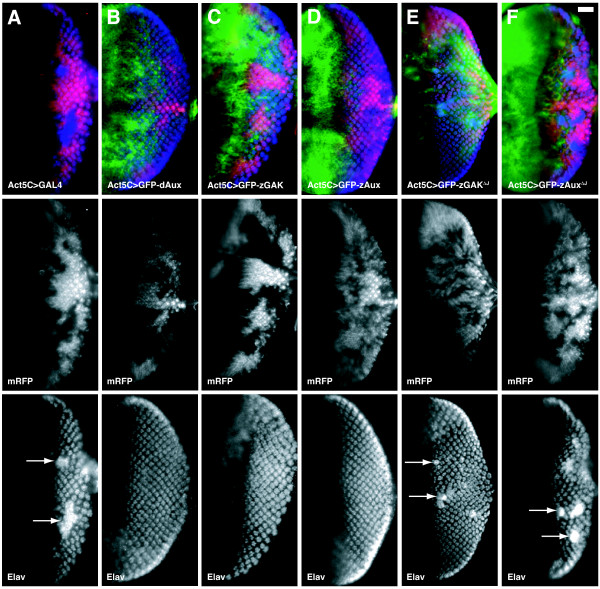
**Both zGAK and zAux can substitute for *Drosophila *auxilin**. Fluorescent micrographs of *Drosophila *eye imaginal discs stained with αElav antibody, which labels the nuclei of the neuronal cells (blue). The expressions of zebrafish and *Drosophila *auxilin genes are shown in green. Homozygous *dAux*^F956^* mutant tissues are indicated by the absence of the membrane-associated mRFP (red). Regions containing excessive Elav-positive cells are indicated by arrows. All the flies carry *Act5C-GAL4*, *UAS-FLP *on the second chromosome, and other relevant genotypes include: (A) *FRT*^*5-5Z3515*^, *dAux*^F956*^/*FRT*^*5-5Z3515*^, *GMR-src-mRFP*, (B) *UAS-dAux-GFP; FRT*^*5-5Z3515*^, *dAux*^F956*^/*FRT*^*5-5Z3515*^, *GMR-src-mRFP*, (C) *UAS-GFP-zGAK*; *FRT*^*5-5Z3515*^, *dAux*^F956*^/*FRT*^*5-5Z3515*^, *GMR-src-mRFP*, (D) *UAS-GFP-zAux*; *FRT*^*5-5Z3515*^, *dAux*^F956*^/*FRT*^*5-5Z3515*^, *GMR-src-mRFP*, (E) *UAS-GFP-zGAK*^Δ*J*^; *FRT*^*5-5Z3515*^, *dAux*^F956*^/*FRT*^*5-5Z3515*^, *GMR-src-mRFP*, and (F) *UAS-GFP-zAux*^Δ*J*^; *FRT*^*5-5Z3515*^, *dAux*^F956*^/*FRT*^*5-5Z3515*^, *GMR-src-mRFP*. Scale Bar, 50 μm.

In wild-type eye discs, regular arrays of photoreceptor clusters could be revealed with αElav antibody, which labels the nuclei of neuronal cells [39]. In contrast, excessive and disorganized Elav-positive cells were seen in *dAux*^*F956X *^mutant tissues (Figure 4A) [33]. This defect of extra Elav-positive cells could be rescued by an *Act5C-GAL4*-driven expression of the full-length *dAux *(Figure 4B) [33]. Similar to *dAux*, both *UAS-GFP-zGAK *and *UAS-GFP-zAux*, when driven by *Act5C-GAL4*, could restore the normal Elav staining pattern in *dAux*^*F956X *^mutant clones (Figure 4C &4D). We have previously shown that the lethality of *dAux *mutants could be rescued by a ubiquitous expression of full-length *dAux *[33]. Similarly, *Act5C-GAL4*-driven *GFP-zGAK *and *GFP-zAux *could both restore the viability of *dAux *mutant flies. These results suggest that zGAK and zAux can both functionally substitute for dAux.

Deletion of the J-domain from *Drosophila *auxilin is known to render them non-functional [32,33]. To ask whether the J-domain is essential for both *zAux *and *zGAK *to rescue *dAux*, *UAS-GFP-zGAK*^DelJ ^and *UAS-GFP-zAux*^DelJ ^were generated. Unlike their full-length counter parts, these J-deletions failed to rescue the extra Elav-positive cell phenotype (Figure 4E &4F) and the lethality, suggesting that, like dAux, the J-domain is critical for the functions of these auxilin family proteins.

### *zGAK *and *zAux *are differentially expressed during embryonic development

**Figure 5:**
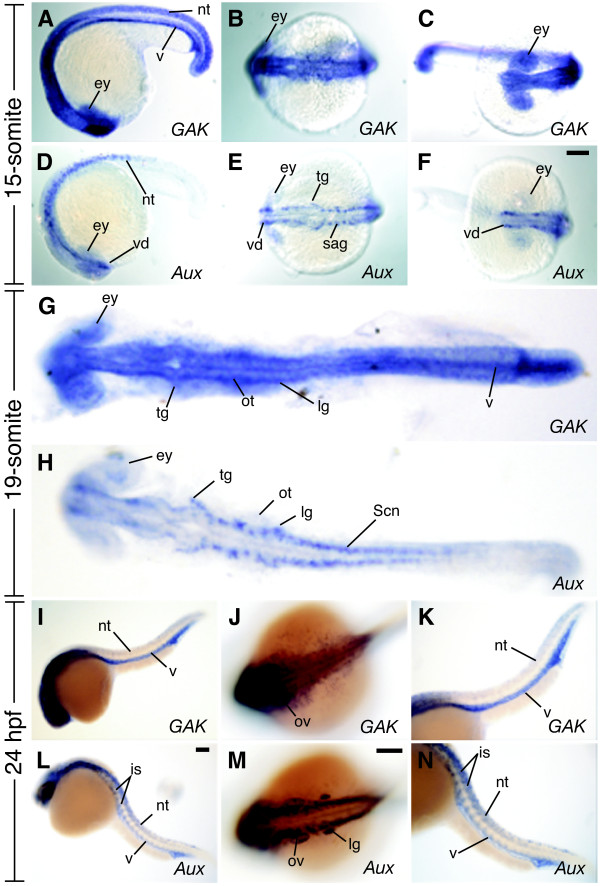
**The expression patterns of *GAK *and *auxilin *during zebrafish embryonic development**. (A, D) Lateral, (B, E) dorsal, and (C, F) anterior views of wild-type embryos at the 15-somite stage. (A-C) *zGAK *is expressed broadly in the hindbrain (B), forebrain and eyes (C). (D-F) *zAux *is expressed mostly in neural tissues as described in the main text. (G, H) Dorsal views of 19-somite stage embryos. (G) *zGAK *is still ubiquitously expressed and (H) *zAux *remains specific to bilateral stripes of neural cells. (I, K, L, N) Lateral and (J, M) dorsal views of 24 hpf embryos. (I-K) *zGAK *is seen in the brain, vasculature and otic vesicles. (L-N) *zAux *remains concentrated in bilateral stripes of neural cells. Panels K and N are close-up views of the posterior regions of the embryos shown in (I) and (L), respectively. In all the images, anterior is to the left, and in all the lateral views, dorsal is up. ey, eye; is, intersomitic vessel; lg, lateral line ganglion; nt, neural tube; ot, otocyte; ov, otic vesicle; sag, statoacoustic ganglion; Scn, Spinal cord neuron; tg, trigeminal ganglion; v, vasculature; vd, ventral diencephalon. Scale Bar, 100 μm.

Although it is known that mammalian *GAK *and *auxilin *are expressed in different tissues [24], their temporal and spatial expressions during development have not been fully investigated. To better understand the requirement of *zGAK *and *zAux*, we examined their expression at various stages of zebrafish development using whole-mount in situ hybridization. Zygotic expressions of *zGAK *and *zAux*, which were also maternally expressed (data not shown), were detected in diverse tissues, but were most prominently associated with neural tissues. Between 8- to 15-somite stages, *zGAK *was broadly expressed in the dorsal region of the embryo, and the expression of *zAux *appeared to be restricted to the bilateral cell clusters on the dorsal side of the embryo (Figure 5A-F). As a control, hybridization using sense probes did not yield detectable signals (data not shown). At the 19-somite stage, *zGAK *still expressed broadly in the entire embryo, whereas *zAux *was predominantly found in hindbrain neurons, spinal chord neurons, and otic vesicles (Figure 5G and 5H). At 24-hour post fertilization (hpf), *zGAK *was enriched in brains, eyes, otic vesicles, and vasculature, and *zAux *expression was detected in hindbrain neurons, spinal chord neurons, otic vesicles and posterior otic capsules (Figure 5I-N). The fact that the expression patterns of *zGAK *and *zAux *did not coincide completely suggests that they have overlapping and distinct functions during development.

### Inhibiting *zGAK *function causes neural-specific cell degeneration

**Figure 6:**
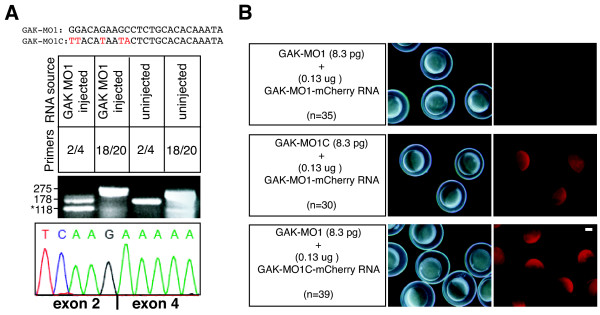
**GAK-MO1 specifically disrupts zGAK splicing**. (A) RT-PCR analysis of total RNA extracted from uninjected and *GAK-MO1 *morphant embryos. From normally spliced *zGAK *mRNA, a reaction with primers complementary to exon 2 and 4 (2/4) would yield a band of 178 bp, and a reaction with primers complementary to exon 18 and 20 (18/20) would yield a band of 275 bp. While the 275 bp band seemed unaffected by GAK-MO1 injection, the level of 178 bp band was reduced and a new band of 118 bp appeared in GAK morphants. Sequence analysis of this 118 bp band showed that exon 2 was spliced into exon 4, resulting in an in-frame deletion in the kinase domain. (B) The target sequences of GAK-MO1 and GAK-MO1C were placed in front of mCherry and cloned into pCS2. In vitro transcribed mRNAs were co-injected with morpholino into one-cell stage embryos. Bright field and fluorescent images of injected embryos at 6 hpf are shown. Scale Bar, 200 μm.

Because of the structural conservation of the GAK genes from *Drosophila *to human, we decided to focus on zGAK function during embryonic development. To inhibit *zGAK*, we designed an antisense morpholino oligonucleotide (GAK-MO1) to disrupt the normal splicing of *zGAK *mRNA (Figure 6A). A control antisense morpholino oligonucleotide (GAK-MO1C), which is a modified sequence of *GAK-MO1 *with five nucleotide changes, was also included. Embryos injected with 8.0ng of GAK-MO1 at one-cell stage (hereafter referred as GAK-MO1 morphants) consistently had smaller eyes, enlarged hindbrain, thinner yolk extension, and weak circulation at 36 to 48 hpf (85%, n = 100, see below). In contrast, injection of the same or higher amount of GAK-MO1C caused no developmental defects in embryos (100%, n = 100). To ensure that the splicing on *zGAK *mRNA was disrupted, RNAs isolated from *GAK-MO1 *morphants at 6 hpf were analyzed by RT-PCR. As shown in Figure 6A, the injection of GAK-MO1 resulted in a transcript lacking exon 3, generating an in-frame deletion in the kinase domain (Figure 1A).

**Figure 7:**
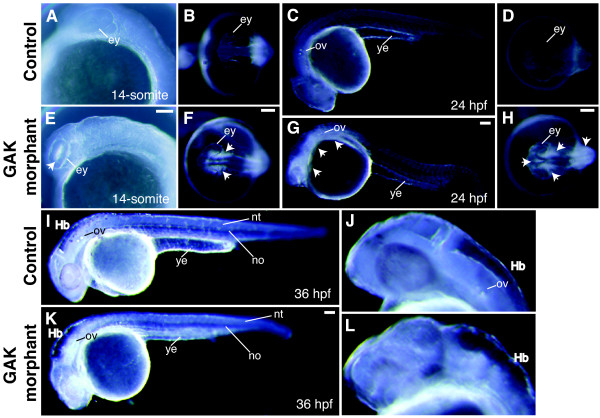
**Cell degeneration phenotype of *GAK *morphant embryos during development**. (A, E) Lateral and (B, F) dorsal views of (A, B) uninjected and (E, F) *GAK *morphant embryos at 14-somite stage. Visible degeneration in the eyes (E) and the forebrain regions (F) of *GAK *morphants is indicated by white arrowheads. (C, G) Lateral and (D, H) dorsal views of (C, D) uninjected and (G, H) *GAK *morphant embryos at 24 hpf. At this stage, the increased cell degeneration within the brain (arrowheads) and thinner yolk extension in *GAK *morphants is apparent. (I-L) Lateral views of (I, J) uninjected and (K, L) *GAK *morphant embryos at 36 hpf. In all the lateral views, anterior is to the left and dorsal is up. ey, eye; Hb, hindbrain; nt, neural tube; no, notochord; ov, otic vesicles; ye, yolk extension. Scale Bar, 100 μm.

Morphologically, the development of *GAK-MO1 *morphants appeared normal until the segmentation stage. At the 10-somite stage, GAK-MO1 morphants displayed cell degeneration in the eyes, which became noticeably opaque (data not shown). At the 14-somite stage, degenerating cells persisted in the eyes, began to appear in the hypothalamic and thalamic regions of the brain and the hindbrain (Figure 7E and 7F), and continued to increase for the remaining duration of somitogenesis. In contrast, these same regions of the uninjected embryos were transparent (Figure 7A and 7B). In some cases, the degeneration had spread to the developing neural tube of GAK-MO1 morphants. It is noteworthy that cell degeneration was only observed in neuroectodermal tissues, but not in non-neural tissues such as the notochord and somites. From 24 to 28 hpf, thinner yolk extension and weak blood circulation were observed in *GAK-MO1 *morphants, as compared to the uninjected embryos at the same stages (Figure 7C, D, G, and 7H). At 36 hpf, the hindbrain and the tectal ventricles of *GAK-MO1 *morphants were enlarged (Figure 7I-L), although these embryos still retained the ability to respond to touch (data not shown).

To ensure that these phenotypes were caused by the disruption of *zGAK*, we tested the binding specificity of GAK-MO1. The complimentary target sequence of GAK-MO1 was placed in front of the mCherry reporter gene [40], and the resulting fusion (*GAK-MO1-mCherry*) was cloned into *pCS2 *(Figure 6C). Similarly, the complimentary sequence for GAK-MO1C was placed in front of mCherry and cloned into *pCS2 *as a control. Using these constructs, *GAK-MO1-mCherry *and *GAK-MO1C-mCherry *RNAs were transcribed in vitro and injected along with either GAK-MO1 or GAK-MO1C morpholinos into one-cell stage embryos. Injected embryos at 6 hpf were then analyzed for the protein expression of mCherry. As shown in Figure 6B, GAK-MO1, but not GAK-MO1C, was capable of blocking the translation of *GAK-MO1-mCherry*. Conversely, GAK-MO1 had no effect on the expression of GAK-MO1C-mCherry. These results demonstrate a high degree of binding specificity and efficacy of GAK-MO1 to its intended target.

To further show that the phenotypes of *GAK-MO1 *morphants were not due to off-target effects, a second morpholino antisense oligonucleotide (GAK-MO2) was designed to block splicing at a different region of the GAK gene (Figure 1A). RT-PCR analysis showed that injection of GAK-MO2 caused the retention of intron 19, resulting in a premature stop codon in the PTEN domain in the *zGAK *transcripts (Figure 1A and Additional file 1: Figure S1A). Embryos injected with 6.0ng of GAK-MO2 exhibited the same phenotypes as those observed with *GAK-MO1 *morphants (90%, n= 85; Additional file 1: Figure S1B and C). Taken together, these results strongly suggest that the developmental defects of *GAK-MO1 *and *GAK-MO2 *morphants resulted from the specific inhibition of *zGAK *function.

### Disrupting *zGAK *function causes excessive neural-specific programmed cell death

**Figure 8:**
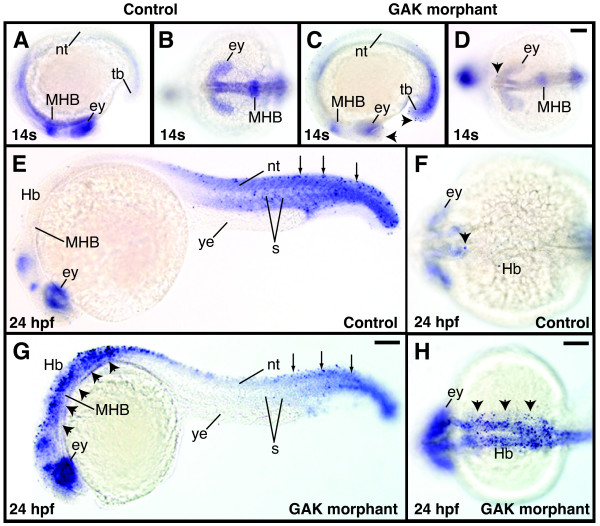
**Programmed cell death in wild-type and *GAK *morphant embryos during development**. (A, C) Lateral and (B, D) dorsal views of TUNEL-stained (A, B) wild-type and (C, D) *GAK *morphant embryos at the 14-somite stage. At this stage, the control embryos display no detectable apoptosis, whereas *GAK *morphants have a low level of programmed cell death (indicated by black arrowheads). (E, G) Lateral and (F, H) dorsal views of TUNEL-stained (E, F) wild-type and (G, H) *GAK *morphants at 24 hpf. While the control animal exhibits a low level of apoptosis in anterior brain and the posterior body regions (arrowheads), *GAK *morphants exhibit a high level of apoptotic cell death in the brain, as well as in the neural tube (arrowheads). However, as compared to the control wild-type embryo, no obvious increase in apoptosis was observed in the posterior region of *GAK *morphants (indicated by arrows). In all the lateral views, anterior is to the left and dorsal is up. ey, eye; Hb, hindbrain; MHB, mid-hindbrain boundary; nt, neural tube; s, somites; tb, tailbud. Scale Bar, 100 m.

To further define the extent and understand the cause of the cell degeneration in *GAK *morphants, we performed in situ TUNEL assay to detect apoptotic cells. At the 14-somite stage, a slight increase in apoptotic cell death was observed in the brain regions of *GAK *morphants, as compared to the control embryos (Figure 8A-D). At 24 hpf, *GAK *morphants displayed a significantly higher level of cell death in the brain, as well as in the neural tube (Figure 8E-H). Similar to the aforementioned cell degeneration, excessive apoptosis in *GAK *morphants was seen only in neural tissues, but not in non-neural tissues. Thus, there appears to be a good correlation in the onset and the extent of these two phenotypes, suggesting that this increase in the programmed cell death is the basis for the cell degeneration in *GAK *morphants.

### Reduction of *zGAK *disrupts the development of specific brain regions

**Figure 9:**
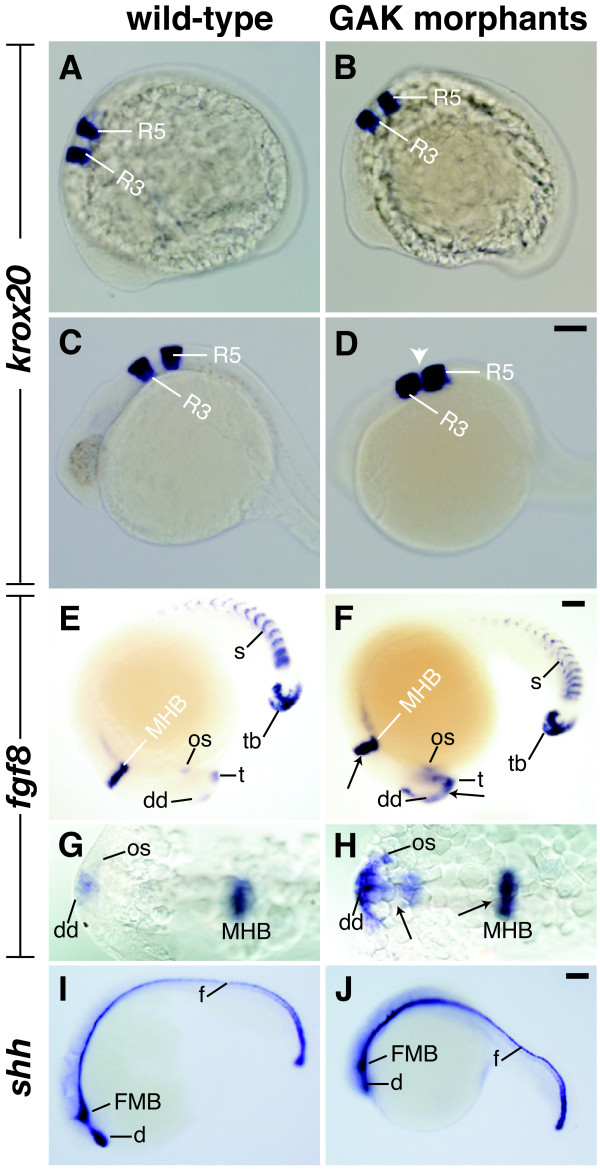
**Expression patterns of regional brain markers in wild-type and *GAK *morphant embryos**. (A-D) *krox20 *expression in (A, C) uninjected and (B, D) *GAK *morphant embryos at (A, B) 12- to 14-somite stage and (C, D) 24 hpf. At 12- to 14-somite stage, *krox20 *expression in the rhombomeres 3 (R3) and 5 (R5) of the control and injected embryos appears comparable. However, at 24 hpf, the spacing between R3 and R5 is dramatically reduced in *GAK *morphants (white arrowhead). (E, F) Lateral and (G, H) dorsal views of *fgf8 *expression in (E, G) wild-type and (F, H) *GAK *morphant embryos at 18- to 20-somite stage. The domains of *fgf8 *expression in the forebrain and mid-hindbrain boundary (MHB) are up-regulated in *GAK *morphants (black arrows). (I, J) *shh *expression in (I) wild-type and (J) *GAK *morphants at the 18-somite stage. In all the lateral views, anterior is to the left and dorsal is up. d, ventral diencephalon; f, floor plate; os, optic stalk; s, somite; t, telencephalon; dd, dorsal diencephalon; tb, tailbud. Scale Bar, 100 μm.

To explore the role of *zGAK *in brain patterning, *GAK *morphants were stained for *krox20, fibroblast growth factor 8 (fgf8) *and *sonic hedgehog (shh)*, whose expressions delineate various regions of the brain. *Krox20*, a zinc finger-containing transcription factor, is expressed specifically in the hindbrain [41]. At the 12-somite stage, the pattern and the level of *krox20 *expression appeared normal in *GAK *morphants (Figure 9A and 9B). At 24 hpf, the pattern of *krox20 *expression in *GAK *morphants remained similar to that seen in wild type, although the two stripes of *krox20 *in the rhombomeres 3 and 5 appeared much closer (Figure 9C and 9D). This suggests that, while the overall patterning of the brain regions is not significantly affected, the rostral hindbrain is reduced in size in the absence of *zGAK *function.

*Fgf8*, expressed in the forebrain, the MHB (midbrain-hindbrain boundary) and other mesodermal derivatives that include somites and tailbud, has been shown to regulate forebrain development and establish the segmental identity of the hindbrain [42]. At the 12-somite stage, the expression of *fgf8 *in GAK morphants appeared to be normal (data not shown). In contrast, GAK morphants at the 18-somite stage exhibited higher levels of fgf8 expression in the MHB, optic stalks and forebrain, suggestive of a requirement for GAK in repressing *fgf8 *expression in these regions (Figure 9E-H). No alteration in *fgf8 *expression in the somites and tailbud regions was observed (Figure 9E and 9F), consistent with the notion that *zGAK *function is required predominantly in neural tissues, but not in non-neural tissues. At the 18-somite stage, *shh *mRNA was detected in the ventral midbrain, hypothalamus, telencephalon and notochord [43]. In contrast to *fgf8*, shh expression appeared normal in the *GAK *morphants at this stage (Figure 9I and 9J).

### Reduction of *zGAK *impairs Notch signaling

**Figure 10:**
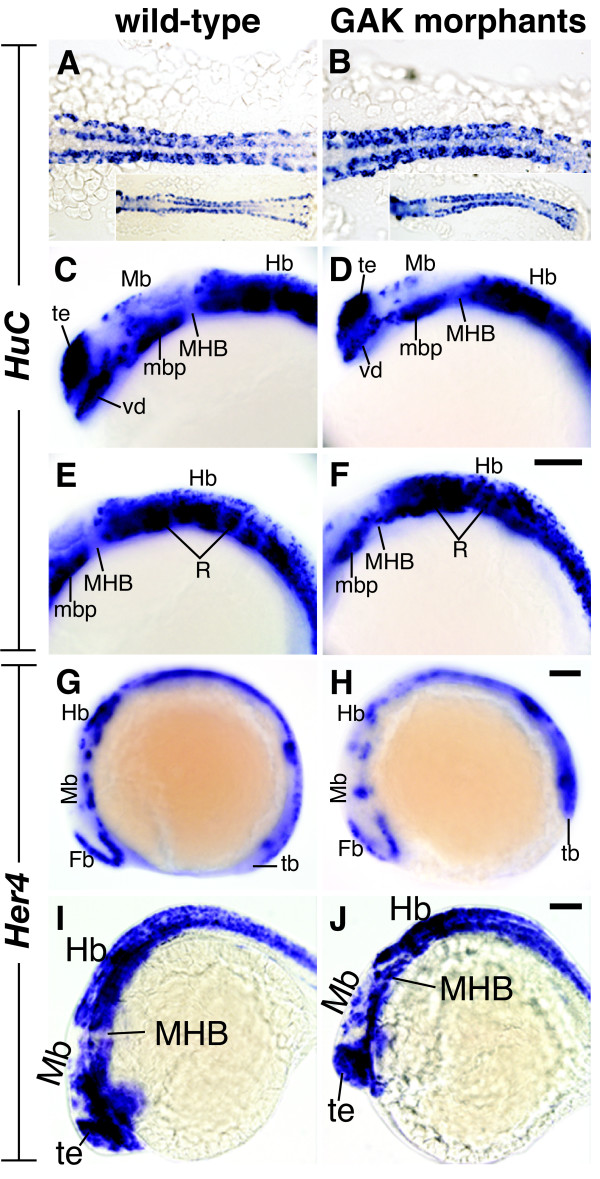
**Expression patterns of *HuC *and *Her4 *in wild-type and *GAK *morphant embryos**. (A, B) Close-up dorsal views of *HuC *expression in (A) wild-type and (B) *GAK *morphant embryos at the 8-somite stage. Micrographs of a lower magnification are shown in the insets. At this stage, more cells appeared to express *HuC *in *GAK *morphant embryos, suggesting the presence of more neural progenitor cells. (C-F) Lateral views of *HuC *expression in the brain regions of (C, E) wild-type and (D, F) *GAK *morphant embryos at 24 to 28 hpf. A comparison of (C) and (D) shows a reduction in *HuC*-positive cells in the forebrain and midbrain in *GAK *morphants. Similarly, a comparison of (E) and (F) shows the disorganization and reduction of *HuC*-positive cells in the hindbrain in *GAK *morphants. (G-J) Lateral views of *Her4 *expression patterns in (G, I) wild-type and (H, J) *GAK *morphants at the (G, H) 8-somite stage or at (I, J) 24 to 28 hpf. At the 8-somite stage, *Her4 *expression in *GAK *morphant embryos was significantly reduced, as compared to the wild type. In contrast, the expression of *Her4 *in the brain of wild-type and *GAK *morphant embryos at 24 to 28 hpf was comparable. In all the panels, anterior is to the left, and in all the lateral views, dorsal is up. Fb, forebrain; Hb, hindbrain; MHB, mid-hindbrain boundary; Mb, midbrain; mbp, midbrain basal plate; R, rhombomeres; tb, tailbud; te, telencephalon; vd, ventral diencephalon. Scale Bar, 100 μm.

To ask if zGAK, like dAux, has a role in Notch signaling, we examined the expression of *HuC *and *Her4 *mRNAs in *GAK *morphants. *HuC*, the zebrafish homolog of *Elav*, is one of the earliest markers for neuronal cells [44]. In 8-somite stage wild-type embryos, *HuC *transcripts are detected in bilateral stripes of neuronal cells along the anterior-posterior axis [45]. In *GAK *morphants at similar stage, although the pattern of expression was similar, more *HuC*-positive cells were seen (Figure 10A and 10B), suggesting that additional cells had adopted neuronal fate. This increase in *HuC *staining persisted in *GAK *morphant embryos at 10-somite stage (Additional file 2: Figure S2A-D). Interestingly, by 24 hpf, *GAK *morphants appeared to have fewer *HuC*-positive cells in the forebrain and midbrain regions (Figure 10C-F). However, it is important to note that the reduction in *HuC*-positive cells at this stage coincides with the onset of the elevated cell death in neural tissues (see above).

*Her4*, a homolog of Drosophila *E(spl) (Enhancer of split) *gene, is a known target of the Notch pathway and participates in specification of neuronal cells [46]. In 8-somite stage wild-type embryos, *Her4 *is expressed in bilateral stripes of cells [46]. Compared to wild type, the level of *Her4 *expression in *GAK *morphants appeared lower (Figure 10G and 10H), suggesting that the output of the Notch pathway was reduced at this stage. Similar to *HuC*, this decrease in *Her4 *expression was still observed in 10-somite stage *GAK *morphant embryos (Additional file 2: Figure S2E-H). At 24 hpf, *Her4 *expression in *GAK *morphants and control embryos was not significantly different (Figure 10I and 10J), suggesting that either GAK is not required for Notch signaling, or GAK and auxilin are redundant for Notch signaling at this stage. Nevertheless, the alterations in the expression of *HuC *and *Her4 *at the 8- and 10-somite stages showed that a reduction of GAK function could impair Notch activity in zebrafish.

## Discussion

Zebrafish, like mammals but not invertebrates, has two distinct auxilin-related genes. Like their mammalian counterparts, zebrafish GAK and auxilin differ in the presence of the N-terminal kinase and their respective expression patterns. zGAK has a kinase domain and is ubiquitously expressed during embryonic development. In contrast, the expression of *zAux*, the ortholog without the kinase domain, appears predominantly in the neuronal cells. There are other differences between the two at the protein sequence level. For instance, zGAK, but not zAux, contains a sequence of FGDL at the amino acid position 950, which matches perfectly to the consensus ψG[PDE][ψLM] (ψ is an aromatic residue). This motif, conserved in mammalian GAKs, has been shown to mediate interactions with AP1 adaptor in Golgi-lysosomal trafficking [31]. Taken together, these structural differences between GAK and auxilin suggest that their molecular functions may have diverged during evolution. It is notable that inhibiting *zGAK *function causes an increase in apoptotic cell death in the neuroectodermal tissues, where GAK and auxilin are both expressed. This inability of *zAux *to compensate for the *zGAK *knockdown would argue that GAK has a function unique from auxilin. However, we cannot formally exclude the possibility that these neural cells may have a higher demand for the functions of *auxilin*-related genes. In this scenario, the functions of both genes would be needed to prevent the onset of apoptosis; therefore, inhibiting GAK alone would be sufficient to induce extra neural cell deaths.

Despite the structural differences between GAK and auxilin, it seems clear that these two paralogs have overlapping molecular functions. Both GAK and auxilin are required for receptor-mediated endocytosis in HeLa cells, indicating that they act in the same process [26]. Consistent with this, we showed that the subcellular localizations of zGAK or zAux in HeLa cells were similar, and over-expression of either in HeLa cells could form clathrin-containing aggregates. More importantly, we showed that over-expression of either zGAK or zAux in *Drosophila *could completely restore the neurogenic defects caused by *dAux*, and this ability to rescue *dAux *absolutely requires their respective J-domains. Together, these results suggest that zGAK and zAux are at least partially redundant.

Unlike zGAK or dAux, zAux does not have the N-terminal Ark kinase. Nevertheless, over-expression of zAux in *Drosophila *could completely restore the defects caused by a strong *dAux *allele. This is not entirely surprising as we have previous shown that over-expression of *dAux*^ΔK^, a *dAux *with its kinase domain deleted, could rescue the dAux phenotype [33]. In fact, over-expression of a fragment consisting of the CBM and J domains alone appears sufficient to restore the function of dAux in Notch [32,33]. However, the kinase domain does have a role in GAK's function in Notch as kinase domain-specific disruptions, by either point mutations [33] or morpholino-induced mis-splicing (MO1, this study), produce *Notch*-like phenotypes. Still, it does appear that, when expressed at a high level, the kinase activity is not required for the functions of auxilin family proteins.

In *Drosophila*, auxilin has been shown to participate in Notch signaling by facilitating ligand internalization [21,22]. Given that mammals and vertebrates have two *auxilin*-related genes, it is not known if either, neither, or both function in the Notch pathway. The similarities in domain structures and expression patterns suggest that GAK is more likely to have a role in Notch. However, while inactivation of *GAK *affects the formation of multiple tissues in mouse [37], it was unclear whether these defects were caused by disrupted Notch signaling. Here we showed that, in *GAK *morphants, the number of *HuC*-positive cells appeared increased, a defect analogous to the neurogenic phenotype. Moreover, we showed that, in *GAK *morphants, the expression of *Her4*, a known Notch target gene, was reduced. Thus, while it is not yet known whether zGAK participates in ligand internalization, these defects in *HuC *and *Her4 *expressions suggest that GAK function is also required for Notch signaling in zebrafish. These results provide the first evidence that the requirement for a GAK-dependent endocytic pathway during Notch signaling is evolutionarily conserved. A requirement of the *mindbomb *E3 ligase and epsin in Notch has also been demonstrated in flies [7,11,12,15,16], fish [9], and mouse [8,47,48], which, along with our analysis of zebrafish GAK, suggests that Notch ligand internalization may rely on the same set of endocytic genes.

It has been demonstrated that a conditional removal of GAK function during mouse brain development causes a significant loss of neural tissues [37], although the mechanism is not known. Likewise, our depletion of GAK function during zebrafish embryonic development results in neural-specific cell degeneration. Using TUNEL staining, we showed that this cell degeneration is caused by increased programmed cell death, suggesting that GAK has a role in preventing the apoptosis of neural cells. Thus, although more *HuC*-positive cells were present in *zGAK*-deficient embryos at the 8- and 10-somite stages, fewer *HuC*-positive cells might be expected at later stages because of cell death. Indeed, this was precisely what we observed, as fewer *HuC*-positive cells were seen in *GAK *morphants at 24 hpf. Interestingly, in *mindbomb *mutant embryos, where Notch ligand endocytosis is impaired, no cell degeneration phenotype was observed (J.S., unpublished data). This, along with our observation that *Her4 *expression was not significantly reduced at 24 hpf, suggests that the role of GAK in maintaining neural cell survival may be Notch-independent. Taken together, our results suggest that *GAK *has at least two distinct functions during the development of neural tissues: an early role in the patterning of neuronal cells and a later role in maintaining the survival of neuronal cells. Furthermore, human GAK has recently been implicated as a susceptibility gene in familial Parkinson disease [49], and the neurodegenerative phenotype observed in GAK morphants certainly supports this conclusion.

It is noteworthy that the phenotypes of the *GAK *morphants bear a strong resemblance to those of the *"spacehead" *class zebrafish mutants [50]. These mutants, isolated from a large-scale screen, are characterized by defects including cell degeneration in the eye and the brain regions, thinner yolk tube, and weak blood circulation [50]. As the genes responsible for most of these mutants have not been determined, the phenotypic similarities suggest that *zGAK *may correspond to one of them. If this is indeed the case, it will provide important clues to understand the functions of these genes in maintaining neuronal cell survival.

## Conclusion

Zebrafish, like mammals but not invertebrates, has two distinct auxilin-related genes, auxilin and GAK. These two genes share some molecular functions, but are not completely redundant, as they are differentially expressed during development. Inhibition of GAK function appears to impair Notch signaling during embryonic neural patterning. This, along with the fact that auxilin has been implicated in *Drosophila *Notch signaling, suggests that the Notch pathway is regulated by a similar set of endocytic factors. In addition, we showed that inhibition of GAK function increases apoptosis in neural tissues, suggesting that GAK has a role in promoting or maintaining the survival of neural cells. As GAK is recently implicated in familial Parkinson disease [49], our results should provide a useful model for further understanding the cause of this neurodegenerative disease.

## Methods

### Embryos and morpholino oligoneucleotides injections

All animal procedures were reviewed and approved by the Purdue Animal Care and Use Committee (PUCAC #06-111-09). Adult fish and embryos were raised and maintained at 28.5°C in system water. Embryos were obtained by natural spawning of adult AB strain zebrafish. *zGAK*-specific antisense morpholino oligonucleotides (GAK-MO1, GAK-MO2, GAK-MO1C, and GAK-MO2C) were purchased from Gene Tools (Philomath, Oregon). At the one-cell stage, each embryo was injected with approximately 1 nl volume of morpholino using a Picospritzer III (Parker Hannifin). Embryos were collected at the appropriate stages [51] and fixed in 4% paraformaldehyde in phosphate-buffered saline (PBS) overnight at 4°C. Fixed embryos were dechorionated, washed 3 times with PBS, and stored in methanol at -20°C.

### *Drosophila *genetics

Flies were raised at 25°C on standard food. Mutant clones of *dAux*^*F956X*^, a strong allele with a nonsense mutation deleting the J-domain, were generated as previously described [33]. For the lethality assay, a trans-heterozygous combination of *dAux*^*F956X*^/*dAux*^*L78H *^was used to avoid potential unrelated lethal mutations in the background. The *dAux*^*L78H *^allele contains a missense mutation in the kinase domain [33]. Immunostaining of eye imaginal discs was performed as previously described [52]. Mouse αElav 9F8A9 (DSHB, Iowa) was used at 1:100.

### Molecular cloning

A 4475 bps-long cDNA containing the entire zGAK ORF in pCCM114 was obtained from OpenBiosystems (ID 2504096). This particular clone has several mutations, including missense mutations at Arg^303^, Tyr^480^, Asp^614^, and a frameshift mutation at Gly^1047^. All were repaired using the QuickChange site-directed mutagenesis kit (Stratagene) and the resulting cDNA was verified by sequencing. For zAux, an Exelixis EST (ID 3410313, OpenBiosystems) containing a partial zAux ORF (missing the N-terminal 696 bps) in pSPORT1 was obtained. A full-length cDNA clone was constructed using RT-PCR products from zebrafish embryonic mRNA and standard cloning techniques, and verified by sequencing.

To generate pCS2-GFP-zGAK and pCS2-GFP-zAux, the entire zGAK and zAux ORFs were fused in frame to the C-terminus of EGFP, and the resulting fusions were cloned into pCS2 as EcoRI-XhoI fragments. To make zGAK^ΔJ ^and zAux^ΔJ^, codons for His^1206 ^of *zGAK *and His^801 ^of *zAux *were changed into stops by site-directed mutagenesis. To express zGAK and zAux in *Drosophila*, GFP-tagged full-length or J-domain-deleted cDNAs were cloned into pUAST [53].

For RT-PCR analysis, total RNAs were extracted from embryos using RNeasy Mini Kit (Qiagen) and the RT-PCR reactions were performed using OneStep RT-PCR Kit (Qiagen). Primers GTATGAGGCCCAGGATTTAGGAAG and GTCAGACTCTTCTTTACTGATGGAC were used to examine the splicing at exon 3. Primers GTGCCCAGAAATGCCTCCACTGTC and GCATAACAGGCTGTCGAACCAGGC were used to examine the splicing at exon 19.

### HeLa cell manipulation and microscopy

HeLa cells were maintained in DMEM, supplemented with 10% Fetal Bovine Serum under standard conditions. To express GFP-zGAK or GFP-zAux, 70% confluent HeLa cells in each 10cm dish were transfected with a 0.5ml cocktail, which contained 10 μg plasmid DNA, 25 μl of Fugene HD (Roche) and serum-free DMEM. Cells were harvested and processed for immunostaining 48 hours after transfection. Mouse αChc (Affinity BioReagents) was used at 1:500, and fluorescently-conjugated secondary antibodies (Molecular Probes) were used at 1:100. All images were collected using Olympus BX61 equipped with a Spinning Disc Confocal unit and processed with Photoshop (Adobe).

### In situ hybridization and TUNEL staining

Whole-mount in situ hybridization was performed using digoxigenin-labeled antisense RNA probes and visualized using anti-digoxigenin Fab fragment conjugated with alkaline phosphatase (Roche) as previously described [54]. Riboprobes were made from DNA templates, which were linearized and transcribed with either SP6 or T7 RNA polymerases. Embryos were processed and hybridized as previously described [54].

Whole-mount in situ TUNEL (terminal deoxynucleotide transferase-mediated dUTP nick-end labeling) was performed using the AP (alkaline phosphatase) In Situ Cell Death Detection Kit (Roche) as previously described [55].

## Authors' contributions

T.B. isolated and analyzed zebrafish *auxilin *and *GAK *cDNA, determined the subcellular localization, and performed the Drosophila rescue experiments. J.L.S. performed the phenotypic analysis of the zebrafish *GAK *morphants. K.K. assisted in the RNA in situ hybridization to determine the expression of zebrafish *auxilin *and *GAK*. H.D. assisted in the molecular cloning of zebrafish *auxilin *and *GAK*. H.C.C. and D.P.S. were responsible for most of the experimental design and the manuscript preparation. All the authors have read and approved the final manuscript.

## Supplementary Material

Additional file 1 **The phenotypes of *GAK-MO2 *morphants are similar to those of *GAK-MO1 *morphants**. (A) RT-PCR analysis of the total RNA from embryos injected with GAK-MO2. Using the RNA prepared from uninjected embryos, a 2/4 reaction (using primers complementary to exon 2 and 4) yielded a band of 178 bp, and an 18/20 reaction yielded a band of 275 bp and a slightly smaller non-specific band. On the other hand, using the RNA prepared from *GAK-MO2 *morphants, the 178 bp band was unaffected. However, the 275 bp band was absent (the non-specific band was unaffected) and a new band of 359 bp appeared. Sequence analysis of this 359 bp band revealed that the injection of GAK-MO2 caused the retention of intron 18, resulting in a frame-shift truncation in the PTEN region (immediately after Lys^679^). (B, C) Lateral views of uninjected and *GAK-MO2 *morphants at 36 hpf. In (B), anterior is to the left and dorsal is up, and in (C), anterior is up and dorsal is to the right. ey, eye; Hb, hindbrain; ov, otic vesicles; ye, yolk extension. Scale Bar, 100 μm. Click here for file

Additional file 2 **Expression patterns of *HuC *and *Her4 *in wild-type and *GAK *morphant embryos at the 10-somite stage**. (A, B) Lateral views of *HuC *expression in (A) wild-type and (B) *GAK *morphant embryos at the 10-somite stage. Similar to the 8-somite stage, more cells appeared to express *HuC *in *GAK *morphant embryos at the 10-somite stage, suggesting the presence of more neuronal cells. (C-D) Close-up top views of *HuC *expression in the brain regions (indicated by brackets in A&B) of (C) wild-type and (D) *GAK *morphant embryos. (E, F) Lateral views of *Her4 *expression patterns in (E) wild-type and (F) *GAK *morphants at the 10-somite stage. At this stage, *Her4 *expression in *GAK *morphant embryos appeared reduced, as compared to the wild type. (G-H) Close-up top views of Her4 expression in the brain regions (indicated by brackets in E&F) of (G) wild-type and (H) *GAK *morphant embryos. In all the panels, anterior is to the left, and in all the lateral views, dorsal is up. Fb, forebrain; Hb, hindbrain; Mb, midbrain; mes, mesencephalon; te, telencephalon; Tg, Trigeminal ganglion; tb, tailbud. Scale Bar, 100 μm. Click here for file
